# Quantum‐Assisted Metrology of Neutral Vitamins in the Gas Phase

**DOI:** 10.1002/anie.201704916

**Published:** 2017-07-28

**Authors:** Lukas Mairhofer, Sandra Eibenberger, Joseph P. Cotter, Marion Romirer, Armin Shayeghi, Markus Arndt

**Affiliations:** ^1^ Faculty of Physics, VCQ University of Vienna Boltzmanngasse 5 1090 Vienna Austria; ^2^ Lyman Laboratory Harvard University Department of Physics 17 Oxford Street Cambridge MA 02138 USA; ^3^ Centre for cold matter Blackett Laboratory Imperial College Prince Consort Road London SW7 2BW UK

**Keywords:** deflectometry, electronic structure, interferometry, matter waves, vitamins

## Abstract

It has recently been shown that matter‐wave interferometry can be used to imprint a periodic nanostructure onto a molecular beam, which provides a highly sensitive tool for beam displacement measurements. Herein, we used this feature to measure electronic properties of provitamin A, vitamin E, and vitamin K1 in the gas phase for the first time. The shift of the matter‐wave fringes in a static electric field encodes the molecular susceptibility and the time‐averaged dynamic electric dipole moment. The dependence of the fringe pattern on the intensity of the central light‐wave diffraction grating was used to determine the molecular optical polarizability. Comparison of our experimental findings with molecular dynamics simulations and density functional theory provides a rich picture of the electronic structures and dynamics of these biomolecules in the gas phase with β‐carotene as a particularly interesting example.

Experimental studies with neutral biomolecules in the gas phase are important because they can be used to assess intrinsic electronic properties of these molecules without perturbation by matrix effects.^1^ In particular, vitamins in the gas phase have recently received renewed theoretical interest,^2^ and these ubiquitous but thermally sensitive particles have been experimentally studied in the gas phase by mass spectrometry^3^ and microwave spectroscopy.^4^ Herein, we utilized the benefits of near‐field quantum interference to measure the optical polarizabilities and electric susceptibilities of molecules in the same setup. We compared experimental data with molecular dynamics (MD) simulations combined with density functional theory (DFT) for α‐tocopherol (vitamin E, C_29_H_50_O_2_), phylloquinone (vitamin K1, C_31_H_46_O_2_), and β‐carotene (provitamin A, C_40_H_56_). These compounds are similar in complexity and mass, but differ in their symmetry, polarity, and thermal folding dynamics. These properties influence their static and optical polarizability as well as their permanent and vibration‐induced electric dipole moment.^5^

Following Louis de Broglie,^6^ quantum mechanics assigns a wave nature to matter, for instance, to the center of mass of entire molecules as well as to the electrons inside.^7^ While electron delocalization is the basis of covalent chemical bonding,^8^ the quantum nature of the center‐of‐mass motion of molecules is less commonly observed as it requires the dedicated preparation of delocalization on the micrometer scale. This can be done in our Kapitza–Dirac–Talbot–Lau interferometer (KDTLI),^9^ which is illustrated in Figure 1. All vitamins are evaporated from a ceramic oven at temperatures between 400 K and 460 K to form a molecular beam in high vacuum. They pass through the interferometer and are detected by electron impact ionization quadrupole mass spectrometry (QMS), about two meters downstream from the source. Thermal fragments are formed but are rejected by the quadrupole mass filter. We select a velocity class of the molecular beam by defining a free‐flight parabola in the gravitational field of the Earth by using three slits. The transmitted velocity distribution is determined by chopping the molecular beam in a pseudo‐random sequence and measuring the time of flight to the detector.^10^ For all vitamins described here, the velocity distribution has a mean value of *v*≈200 m s^−1^ with a spread of about 45 % (FWHM). This corresponds to de Broglie wavelengths *λ*_dB_=*h*/*mv* of 3–6 pm, where *h* is Planck's constant, and *m* is the mass of individual molecules. The wavelength *λ*_dB_ is almost three orders of magnitude smaller than the size of each molecule. However, at the position of the second grating, the center‐of‐mass wave function is delocalized across the molecular beam over more than one million *λ*_dB_.

**Figure 1:**
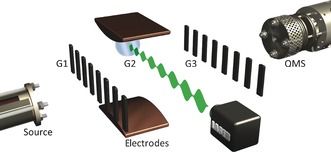
A near‐field matter‐wave interferometer with a high‐voltage deflection electrode. The effusive source emits a beam of vitamins, which diffracts at the gratings G1, G2, and G3. G1 and G3 are nanomechanical gratings (black), G2 is an optical grating (green). The deflection electrode (brown) displaces the observed interference fringes. The quadrupole mass spectrometer (QMS) ionizes and mass‐selects the molecules.

The setup is as follows: The three gratings G1, G2, and G3 have an equal period of *d=*266 nm and are positioned at equal distances along the molecular beam. G1 and G3 are machined into 190 nm thick silicon nitride. The first grating acts as an array of point‐like sources with a width of *s*=110 nm. It follows from Heisenberg's uncertainty relation that each molecule passing through any of the slits of G1 acquires a transverse momentum uncertainty of Δ*p*≥*h*/Δ*x*=*h*/*s*. This delocalization increases linearly with the distance behind G1. When the molecules arrive at the second grating, *L*=10.5 cm downstream from G1, the indeterminacy of the molecular position, that is, their transverse coherence,^11^ has grown to 2*λ*_dB_ *L*/*s*≈1 μm such that it covers several periods of G2. The diffraction grating G2 is an optical standing wave with a period of *λ*_L_/2 that is obtained by retro‐reflecting a laser beam with a wavelength of *λ*_L_ = 532 nm from a mirror. Each molecule, with an optical polarizability $\alpha_{opt}\left(\omega \right)$ , passing through the electric field *E* of G2 experiences an electric dipole potential *W*_opt_=−*α*_opt_(*ω*)*E* ^2^(*x*)/4, which modulates the de Broglie phase of the molecular center‐of‐mass wave function. Free evolution of the matter wave behind G2 leads to the formation of a periodic molecular density distribution. Close to multiples of the Talbot length *L*_T_=*d* ^2^/*λ*_dB_, this pattern is an image of the grating of period *d*.

This interferometer concept is common in optics,^12^ and has been demonstrated in atom optics^13^ as well as medical X‐ray imaging.^14^ Recently, it has been used to reveal the wave nature of molecules as large as 10 000 amu.^15^ Herein, we used this method to study the electronic properties of neutral vitamins in the gas phase. The interference pattern is detected by scanning the nanomechanical mask G3 over the molecular beam, another 10.5 cm behind G2. When the molecular fringes and the grating G3 are in phase, the number of molecules arriving at the spectrometer *S*(*x*) is maximized. The fringe visibility of the sine fit to the data is defined as *V=*(*S*_max_−*S*_min_)/(*S*_max_+*S*_min_). It depends on the optical polarizability *α*_opt_(*ω*) 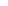 and the absorption cross section *σ*(*ω*) of the molecules as well as the laser intensity in the center of the Gaussian light beam $I=2P/\pi {\;w}_{x}w_{y}$ with the horizontal and vertical waists $w_{x}$ 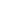 and $w_{y}$ .

High‐visibility interference patterns were observed for all three vitamins. In Figure 2, we show a typical high‐contrast interferogram of β‐carotene, which constitutes clear evidence for the quantum nature of its motional state.^16^ The dependence of the fringe visibility on the laser power *P* allowed us to determine the optical polarizability.^17^ Typical *V*(*P*) curves for α‐tocopherol and phylloquinone are shown in Figure 3. A molecule may also absorb a photon in transit through the diffracting laser field. This is an additional matter‐wave beam splitting mechanism, which also modulates the fringe visibility.^10^ A correct interpretation of the *V*(*P*) curve (Figure 3) thus hinges on good knowledge of the laser intensity in G2, which we have calibrated in situ by using C_60_ molecules.^16^

**Figure 2:**
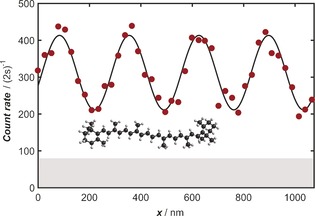
Molecular interference pattern of β‐carotene. The data points (red circles) show the number of detected molecules as a function of the transverse position of grating G3. We observed a sinusoidal variation in the number of counts, whose high amplitude constitutes clear evidence for quantum interference. The solid line is a sinusoidal fit to the data, from which we extracted a fringe visibility of *V*=32±2 %. The gray area highlights the dark counts.

**Figure 3:**
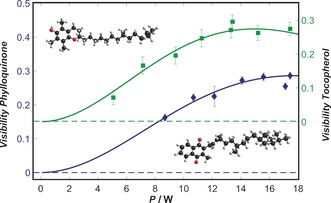
Experimental interference fringe visibilities of phylloquinone (blue diamonds) and α‐tocopherol (green squares) as a function of the diffracting laser power in G2. In the interaction region, the horizontal waist $w_{x}$ 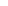 was 20 μm and the vertical waist was 920 μm. The error bars show the uncertainties in the visibility resulting from an error propagation of the amplitude and offset of the sine curve using 68 % confidence intervals for the sine fit.

The nanoscale period of the molecular density pattern enables high‐resolution molecule deflectometry: Any transverse force acting on the molecular beam will displace the fringes by a distance Δ*x*. By using a tailored pair of electrodes, we created a homogeneous force close to the second grating that shifts the interference fringes^18^ by $\Delta x=K\left(\frac{\chi }{m}\right)\frac{\partial }{\partial_{x}}E_{d}^{2}\frac{1}{v^{2}}\;$ . It grows with the electric susceptibility to mass ratio (χ /*m*), the gradient of the square of the deflection field $E_{d}$ , and inversely with the square of the molecular velocity *v*. The geometrical factor *K* contains information about the electrode geometry and position, and was here calibrated using C_60_ molecules. The deflection Δx follows the same law as that found in classical deflectometry^19^ but quantum interference adds the nanometric fine structure, which is valuable for achieving a spatial resolution of the shift on the order of 10 nm.

In Figure 4, the molecular fringe deflection for α‐tocopherol and phylloquinone in an electric field of varying strength is shown. For rigid, non‐polar molecules, the static polarizability can be directly extracted from such deflection measurements. In most vitamins, however, molecular vibrations induce fluctuations of the squared electric dipole moment $\mu^{2}$ whose thermal average ⟨$\mu^{2}\rangle$ contributes to the total electric susceptibility^20^ through the van Vleck relation $\chi =\alpha_{stat}+\left\langle \mu^{2}\right\rangle /3k_{B}T$ , where $k_{B}$ is the Boltzmann constant.

**Figure 4:**
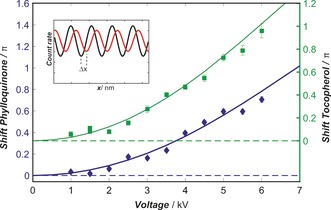
Molecular fringe displacement of phylloquinone (blue diamonds, left scale) and α‐tocopherol (green squares, right scale) as a function of the deflection voltage. As expected, Δ*x* depends quadratically on the voltage. The axes of the two curves are offset vertically for clarity. The velocities were 180 m s^−1^ for α‐tocopherol and 195 m s^−1^ for phylloquinone. The inset sketches the molecular fringe position for two deflection voltages (red=6 kV, black=1 kV). The error bars are 68 % confidence intervals for the relative phase of the two sinusoidal interference curves. At high voltage, the uncertainty increases because the fringe shift and visibility are sensitive to the finite velocity spread of the molecular beam. A shift of π corresponds to a beam displacement of half a grating period.

To extract the electronic properties from our experiments, we compared the data to the results of theoretical calculations. The center‐of‐mass motion is described by a well‐established quantum formalism in phase space.^16^ In the absence of radiation, collisions, and other interactions with the environment, the internal degrees of freedom are decoupled from the molecular center‐of‐mass motion and are described by a combined MD and DFT approach. A full quantum treatment of the internal states is conceivable,^21^ but not necessary for our present study.

Here, we sampled the molecular configurations by MD simulations to account for the flexibility of the vitamins and computed their static and optical polarizabilities (at 532 nm) using the coupled perturbed Kohn–Sham method as well as the electric dipole moments for subsequent time steps by DFT. The conformational space was scanned by an MD simulation using the LAMMPS package^22^ with CHARMM^23^ force field parameters obtained from the multipurpose atom typer for CHARMM.^24^ During the MD simulation, a single molecule was propagated over 100 ns (after an equilibration run of 10 ns) in time steps of 1 fs at the respective experimental oven temperatures of around 450 K controlled by a Nosé–Hoover thermostat^25^ with a relaxation time of 0.1 ps. Assuming that the MD time evolution of a single molecule in vacuum covers a sufficiently large conformational phase space, a single time sequence samples a statistically representative ensemble of conformations in the hot molecular beam. Short ab initio molecular dynamics (AIMD) simulations using NWChem^26^ at the PBE0/3–21G level of theory^27^ over 50 ps indicate that the CHARMM force field is a reasonable approximation for our high‐temperature simulations.

The molecular structure was extracted from the MD simulation every 2 ns and fed into DFT calculations at the CAM‐B3LYP/Def2TZVP level of theory^28^ using the Gaussian 09 program package.^29^ The range‐separated hybrid exchange correlation functional CAM‐B3LYP has been shown to perform well for calculations of electronic (hyper)polarizabilities of organic compounds.^30^

Figure 5 displays the electronic parameters for α‐tocopherol. Simulation data for the other two vitamins are compiled in the Supporting Information. Conformational changes occur on the picosecond scale. Even under vigorous fluctuations, the optical and static polarizabilities remain constant within a few percent, while the dipole moment fluctuates by up to 400 % from peak to peak when sampled on the nanosecond scale. Such calculations allowed us to determine the van Vleck susceptibility *χ*, which is compared with our measurements in Table 1. All computed values represent averages over the DFT calculations for the configurational space sampled by MD simulations. The molecular dynamics simulations suggest that all‐*trans*‐β‐carotene is more rigid than the other two vitamins in its ground state and also non‐polar. However, modeling shows that β‐carotene can develop a non‐zero average dipole moment and may undergo thermally induced *cis*–*trans* isomerization in the gas phase. In solution,^31^ this transition has been observed at temperatures as low as 350 K. Such *cis* geometries are therefore expected to contribute to the experimental results. Furthermore, spectra in solution indicate that β‐carotene exhibits strong wavelength shifts even in moderate electric fields,^32^ and its optical polarizability should therefore depend on the field.

**Figure 5:**
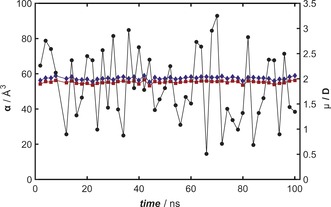
Calculated electronic properties of α‐tocopherol. Evolution of the static (*α*_stat_, red squares) and optical (*α*_opt_ (532 nm), blue diamonds) polarizability as well as the electric dipole moment (*μ*, black circles) during the MD simulation displayed in 2 ns steps. The DFT calculations along the MD trajectory were performed at the CAM‐B3LYP/Def2TZVP level of theory.

**Table 1:** Molecular electronic properties from experiment and theory.^[a]^

Vitamin	E α‐tocopherol	K1 phylloquinone	Pro‐A β‐carotene
Sum formula	C_29_H_50_O_2_	C_31_H_46_O_2_	C_40_H_56_
*T* [K]	400±5	450±5	460±5
Mass [amu]	430.7	450.7	536.9
			
⟨*μ*⟩^[b]^ [D]	1.8±0.1 1.8±0.1	1.1±0.1 1.1±0.1	1.3±0.1 1.3±0.1
			
⟨*α*_stat_⟩^[b,c]^ [Å^3^]	54±1 54±1	58±1 59±1	211±4 229±3
			
⟨*α*_opt_⟩^[b,c]^ [Å^3^]	56±1 56±1 58±5	62±1 61±1 52±7	−152±11 −107±4 (−)83±10
			
*χ* ^[b,c]^ [Å^3^]	78±3 78±3 80±8	65±1 66±1 80±10	229±4 240±3 n.a.

[a] The theoretical values represent a thermal average at the temperature of the experiment. The computational results refer to the mean over all considered MD steps. We estimated the experimental errors by testing the robustness of the result against the combined standard deviations of the contributing factors (see the Supporting Information). [b] First row: CAM‐B3LYP/Def2TZVP; second row: PBE0/Def2TZVP; third row: experiment. [c] Converted into SI units by multiplying with $4\pi \epsilon_{0}$ .

It is surprising that the MD‐averaged optical polarizability of all‐*trans*‐β‐carotene exhibits a negative sign, even though the diffraction laser is red‐detuned to the nearest expected dipole‐allowed transition at around 440 nm. Although the experiment is insensitive to the sign of $\alpha_{opt}$ (532 nm), we have cross‐checked its value using the global hybrid PBE0 functional. While for phylloquinone and α‐tocopherol, PBE0 yields very similar predictions for all electronic properties, β‐carotene is again a special case as the optical polarizability maintains the negative sign but its value changes from −152 Å^3^ (CAM‐B3LYP) to −107 Å^3^ (PBE0). This is not surprising as the polarizability spectra are sensitive to the percentage of Hartree–Fock exchange.^33^ This lower value is also closer to the experimental result of |83±10|Å^3^.

To further elucidate the origin of the sign of $\left\langle \alpha_{opt}\right\rangle$ at the wavelength of the grating laser (532 nm), we calculated optical polarizabilities and spectra by time‐dependent DFT both for the vibrational ground state with inversion symmetry and for distorted carotene geometries. Whereas $\alpha_{opt}$ (532 nm) is positive for the inversion‐symmetric geometry, it exhibits a negative value for distorted structures. This correlates with the calculated optical response: We found an intense dipole transition in the range of 600–650 nm for distorted carotene but not for its ground state. With respect to this transition, the grating laser is blue‐detuned, explaining the negative sign of $\left\langle \alpha_{opt}\;\left(532\;nm\right)\right\rangle$ . Our computation is consistent with recent experiments showing that this transition is accessible in two‐photon processes^34^ and by near‐edge X‐ray absorption combined with UV photoelectron spectroscopy.^35^

In summary, we have shown that quantum‐interference‐assisted metrology opens a window for determining electrical, optical, and dynamical properties of biomolecules in a single comprehensive setting. We have shown this here for the three (pro)vitamins α‐tocopherol, phylloquinone, and β‐carotene. We observed good agreement with computational chemistry and also found that the fully conjugated electronic structure of β‐carotene raises a number of interesting questions. Future studies in molecule interference shall address these also by using highly sensitive single‐photon recoil spectroscopy at around 640 nm.^36^ Sources of internally cold molecules^37^ will allow further elucidation of the role of conformations, which can be supported by more elaborate AIMD simulations. Matter‐wave assisted metrology thus constitutes an interesting link between quantum optics and chemistry. It can be readily extended to magnetic, optical, and collisional properties, and thus help benchmarking computational models of complex biomolecules.

## Conflict of interest

The authors declare no conflict of interest.

## Supporting information

As a service to our authors and readers, this journal provides supporting information supplied by the authors. Such materials are peer reviewed and may be re‐organized for online delivery, but are not copy‐edited or typeset. Technical support issues arising from supporting information (other than missing files) should be addressed to the authors.

Supplementary

## References

[1a] W. Caminati , Angew. Chem. Int. Ed. 2009, 48, 9030–9033; 10.1002/anie.200902993 19856354

[1b] M. F. Jarrold , Annu. Rev. Phys. Chem. 2000, 51, 179–207; 11031280 10.1146/annurev.physchem.51.1.179

[1c] V. Gabelica , Nucleic Acids in the Gas Phase, Springer, Heidelberg, 2014;

[1d] T. Meyer , V. Gabelica , H. Grubmüller , M. Orozco , WIREs Comput. Mol. Sci. 2013, 3, 408–425.

[2a] F. Abyar , H. Farrokhpour , RSC Adv. 2014, 4, 35975;

[2b] H. Dossmann , A. Schwarzenberg , D. Lesage , M. Perot-Taillandier , C. Afonso , B. Cunha de Miranda , G. A. Garcia , J. Phys. Chem. A 2014, 118, 11185–11192. 25340309 10.1021/jp507050y

[3] D. A. Volmer , L. R. Mendes , C. S. Stokes , Mass Spectrom. Rev. 2015, 34, 2–23. 24318020 10.1002/mas.21408

[4] I. Peña , A. M. Daly , C. Cabezas , S. Mata , C. Bermúdez , A. Niño , J. C. López , J.-U. Grabow , J. L. Alonso , J. Phys. Chem. Lett. 2012, 4, 65–69. 26291213 10.1021/jz301947g

[5] I. Compagnon , R. Antoine , D. Rayane , M. Broyer , P. Dugourd , Phys. Rev. Lett. 2002, 89, 253001. 12484879 10.1103/PhysRevLett.89.253001

[6] L. De Broglie , Nature 1923, 112, 540.

[7] M. Arndt , O. Nairz , J. Voss-Andreae , C. Keller , G. van der Zouw , A. Zeilinger , Nature 1999, 401, 680–682. 18494170 10.1038/44348

[8] W. Kutzelnigg , Angew. Chem. Int. Ed. Engl. 1973, 12, 546–562;

[9a] S. Gerlich , L. Hackermüller , K. Hornberger , A. Stibor , H. Ulbricht , M. Gring , F. Goldfarb , T. Savas , M. Müri , M. Mayor , M. Arndt , Nat. Phys. 2007, 3, 711–715;

[9b] K. Hornberger , S. Gerlich , P. Haslinger , S. Nimmrichter , M. Arndt , Rev. Mod. Phys. 2012, 84, 157–173.

[10] J. P. Cotter , S. Eibenberger , L. Mairhofer , X. Cheng , P. Asenbaum , M. Arndt , K. Walter , S. Nimmrichter , K. Hornberger , Nat. Commun. 2015, 6, 7336. 26066053 10.1038/ncomms8336 PMC4477035

[11] A. D. Cronin , J. Schmiedmayer , D. E. Pritchard , Rev. Mod. Phys. 2009, 81, 1051–1129.

[12] K. Patorski in Progress in Optics {XXVII} (Ed.: E. Wolf), Elsevier, Amsterdam, 1989, pp. 2–108.

[13] J. F. Clauser , S. Li , Phys. Rev. A 1994, 49, R2213.

[14] F. Pfeiffer , M. Bech , O. Bunk , P. Kraft , E. F. Eikenberry , C. Bronnimann , C. Grunzweig , C. David , Nat. Mater. 2008, 7, 134–137. 18204454 10.1038/nmat2096

[15] S. Eibenberger , S. Gerlich , M. Arndt , M. Mayor , J. Tüxen , Phys. Chem. Chem. Phys. 2013, 15, 14696–14700. 23900710 10.1039/c3cp51500a

[16] K. Hornberger , S. Gerlich , H. Ulbricht , L. Hackermüller , S. Nimmrichter , I. Goldt , O. Boltalina , M. Arndt , New J. Phys. 2009, 11, 043032.

[17] L. Hackermüller , K. Hornberger , S. Gerlich , M. Gring , H. Ulbricht , M. Arndt , Appl. Phys. B 2007, 89, 469–473.

[18a] S. Gerlich , M. Gring , H. Ulbricht , K. Hornberger , J. Tüxen , M. Mayor , M. Arndt , Angew. Chem. Int. Ed. 2008, 47, 6195–6198; 10.1002/anie.200801942 18624315

[18b] M. Gring , S. Gerlich , S. Eibenberger , S. Nimmrichter , T. Berrada , M. Arndt , H. Ulbricht , K. Hornberger , M. Müri , M. Mayor , M. Böckmann , N. L. Doltsinis , Phys. Rev. A 2010, 81, 031604.

[19a] P. Dugourd , I. Compagnon , F. Lepine , R. Antoine , D. Rayane , M. Broyer , Chem. Phys. Lett. 2001, 336, 511–517;

[19b] W. A. de Heer , V. V. Kresin in Handbook of Nanophysics (Ed.: K. D. Sattler), CRC, Boca Raton, 2011, pp. 10/11–13;

[19c] S. Heiles , R. Schäfer , Dielectric Properties of Isolated Clusters Beam Deflection Studies, Springer, Amsterdam, 2014.

[20] J. H. V. Vleck , The theory of electric and magnetic susceptibilities, Oxford University Press, London, 1965.

[21] M. C. Böhm , R. Ramirez , J. Schulte , Chem. Phys. 1998, 227, 271–300.

[22] http://lammps.sandia.gov/.

[23] B. R. Brooks , R. E. Bruccoleri , B. D. Olafson , D. J. States , S. Swaminathan , M. Karplus , J. Comput. Chem. 1983, 4, 187–217.

[24] J. D. Yesselman , D. J. Price , J. L. Knight , C. L. Brooks , J. Comput. Chem. 2012, 33, 189–202. 22042689 10.1002/jcc.21963 PMC3228871

[25] W. G. Hoover , Phys. Rev. A 1985, 31, 1695–1697. 10.1103/physreva.31.1695 9895674

[26] M. Valiev , E. J. Bylaska , N. Govind , K. Kowalski , T. P. Straatsma , H. J. J. Van Dam , D. Wang , J. Nieplocha , E. Apra , T. L. Windus , W. A. de Jong , Comput. Phys. Commun. 2010, 181, 1477–1489.

[27a] C. Adamo , V. Barone , J. Chem. Phys. 1999, 110, 6158–6170;

[27b] J. S. Binkley , J. A. Pople , W. J. Hehre , J. Am. Chem. Soc. 1980, 102, 939–947.

[28a] T. Yanai , D. P. Tew , N. C. Handy , Chem. Phys. Lett. 2004, 393, 51–57;

[28b] F. Weigend , R. Ahlrichs , Phys. Chem. Chem. Phys. 2005, 7, 3297–3305. 16240044 10.1039/b508541a

[29] M. J. Frisch, G. W. Trucks, H. B. Schlegel, G. E. Scuseria, M. A. Robb, J. R. Cheeseman, J. A. J. Montgomery, T. Vreven, K. N. Kudin, J. C. Burant, J. M. Millam, S. S. Iyengar, J. Tomasi, V. Barone, B. Mennucci, M. Cossi, G. Scalmani, N. Rega, G. A. Petersson, H. Nakatsuji, M. Hada, M. Ehara, K. Toyota, R. Fukuda, J. Hasegawa, M. Ishida, T. Nakajima, Y. Honda, O. Kitao, H. Nakai, M. Klene, X. Li, J. E. Knox, H. P. Hratchian, J. B. Cross, C. Adamo, J. Jaramillo, R. Gomperts, R. E. Stratmann, O. Yazyev, A. J. Austin, R. Cammi, C. Pomelli, J. W. Ochterski, P. Y. Ayala, K. Morokuma, G. A. Voth, P. Salvador, J. J. Dannenberg, V. G. Zakrzewski, S. Dapprich, A. D. Daniels, M. C. Strain, O. Farkas, D. K. Malick, A. D. Rabuck, K. Raghavachari, J. B. Foresman, J. V. Ortiz, Q. Cui, A. G. Baboul, S. Clifford, J. Cioslowski, B. B. Stefanov, G. Liu, A. Liashenko, P. Piskorz, I. Komaromi, R. L. Martin, D. J. Fox, T. Keith, M. A. Al-Laham, C. Y. Peng, A. Nanayakkara, M. Challacombe, P. M. W. Gill, B. Johnson, W. Chen, M. W. Wong, C. Gonzalez, J. A. Pople, Revision A.1 ed., Gaussian Inc., Wallingford, CT, **2009**.

[30a] M. J. G. Peach , E. I. Tellgren , P. Sałek , T. Helgaker , D. J. Tozer , J. Phys. Chem. A 2007, 111, 11930–11935; 17963369 10.1021/jp0754839

[30b] P. A. Limacher , K. V. Mikkelsen , H. P. Luthi , J. Chem. Phys. 2009, 130, 194114. 19466828 10.1063/1.3139023

[31] M. Marx , Food Chem. 2003, 83, 609–617.

[32] H. A. Frank , A. Young , G. Britton , R. J. Cogdell , The photochemistry of carotenoids, Vol. 8, Springer Science & Business Media, New York, 2006.

[33] A. M. Ferrari , R. Orlando , M. Rerat , J. Chem. Theory Comput. 2015, 11, 3245–3258. 26575761 10.1021/acs.jctc.5b00199

[34] P. J. Walla , P. A. Linden , C.-P. Hsu , G. D. Scholes , G. R. Fleming , Proc. Natl. Acad. Sci. USA 2000, 97, 10808–10813. 10984512 10.1073/pnas.190230097 PMC27105

[35] H. S. M. Beck , D. Leupold , B. Winter , D. Pop , U. Vogt , C. Spitz , Biochim. Biophys. Acta Bioenerg. 2001, 1506, 260–267. 10.1016/s0005-2728(01)00226-2 11779559

[36] S. Eibenberger , X. Cheng , J. P. Cotter , M. Arndt , Phys. Rev. Lett. 2014, 112, 250402. 25014795 10.1103/PhysRevLett.112.250402

[37] D. Patterson , J. M. Doyle , Phys. Chem. Chem. Phys. 2015, 17, 5372–5375. 25611165 10.1039/c4cp03818e

