# Supplementary material for: Quantum‐Assisted Metrology of Neutral Vitamins in the Gas Phase
Source: Angew Chem Int Ed Engl. 2017 Jul 28;56(36):10947–51. doi: 10.1002/anie.201704916 (PMC5582605; doi:10.1002/anie.201704916)
Supplement: Supplementary file 1 — Supplementary [file ANIE-56-10947-s001.pdf]

Angewandte Chemie
Eine Zeitschrift der Gesellschaft Deutscher Chemiker

Supporting Information

Quantum-Assisted Metrology of Neutral Vitamins in the Gas Phase

*Lukas Mairhofer, Sandra Eibenberger, Joseph P. Cotter, Marion Romirer, Armin Shayeghi, and Markus Arndt**

anie_201704916_sm_miscellaneous_information.pdf

a) Source and detection

All vitamins are sublimated/evaporated in a ceramic oven with an aperture of 0.2 x 2 mm that was filled with about 200 mg of the material. This allows stable measurements of about 4 hours per source loading. The emitted beam is well approximated by a Maxwell-Boltzmann distribution with a small forward offset velocity. At temperatures between 400 K and 450 K the most probable velocity is then around 200 m/s.

We use a random chopper in combination with time-resolved detection to measure the time-of-flight distribution of the molecular beam. The chopper consists of a disc with a pseudo-random pattern of 64 openings and spins at 15 Hz, resulting in an effective chopping frequency of 980 Hz.
The molecules are detected using electron impact ionization (at 70 eV) and quadrupole mass spectrometry (QMS Extrel CMS). To maximize transmission, the QMS is operated in low resolution mode and the counts are maximized by setting the conversion dynode voltage to 9 kV. Thermal and impact-induced fragments are rejected in the mass filter to ensure we only detect intact parent molecules.

b) Optical polarizabilities

Calibration of the laser beam waist

A correct interpretation of the V(P) curve (Figure 3) hinges on a precise knowledge of the laser intensity in G2. The available laser power in G2 and the molecular polarizability allows observing the first maximum for α-tocopherol and β-carotene but not fully for phylloquinone.

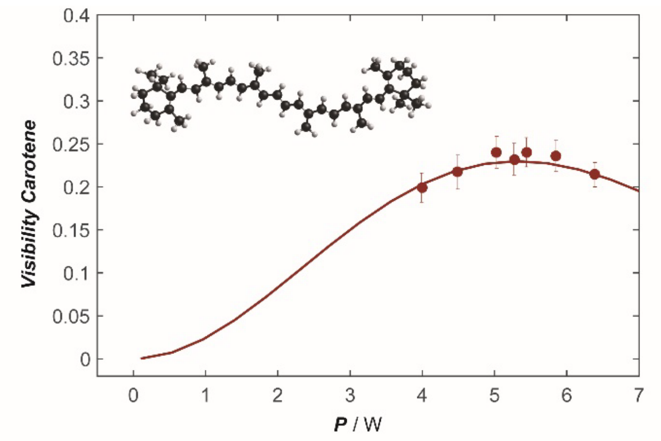

Figure S1. Interference fringe visibility of β-carotene as a function of the diffraction grating laser power in G2. The maximum contrast in this curve of about 23% is lower than that in Figure 2 of the main text, because of a different grating alignment.

Laser power and beam diameter were calibrated with 5% accuracy outside the vacuum chamber before the beam was focused by an f = 100 mm cylindrical lens onto the interferometer mirror *in vacuo*. Changes in the optical polarizability and laser power calibration shift the $V(P)$ curve horizontally. Additional optical absorption reduces the effective interference contrast. This may be mimicked by imperfect alignment of the interferometer as well as by dephasing noise[1], thermal[2] or collisional decoherence[3]. We have used the well-known C_{60} molecules to calibrate any possible experimental visibility reduction as well as the optical beam parameters by comparing the observed C_{60} interference contrast with the theoretical model[4]. The vertical waist of the light grating is calculated from the horizontal position of the maximum of the V(P) curve. Using the C_{60} polarizability $\alpha_{opt} = 4\pi\varepsilon_0 \times 87(10)$ Å^3 and optical absorption cross section $\sigma_{opt}(532nm) = 2.9\times10^{-18} cm^2$ we confirm a waist of 440 µm for the polarizability-measurement and interference of β-carotene and 920 µm for all other measurements.

c) Electric susceptibilities

Measurement of the fringe shift

In our setup an interference pattern is obtained by scanning the third grating transversely over the molecular beam. To determine the field-dependent fringe shift and from this the molecular susceptibility, we record two interference patterns at the same time, one for the reference voltage of 1 kV and one for the deflection voltage. At each position of the third grating G_3 we switch between the two voltages and record the respective number of molecules arriving in the detector. We have measured in a rising and falling voltage sequence to exclude potential residual systematics.

Calibration of the electric deflection field

The proportionality factor *K* in our electric deflection experiments depends on the electrode and the molecular beam geometry and can be calibrated with an atom or molecule of known polarizability. Here we used the known static polarizability of C_{60} to determine K=1924(78) for identical conditions as used for the vitamins K and E (see Figure S1). We plot the shift in units of the fringe phase $\Delta\varphi = 2\pi \cdot \Delta x/d$. A shift of π corresponds to half a period of an interference pattern.

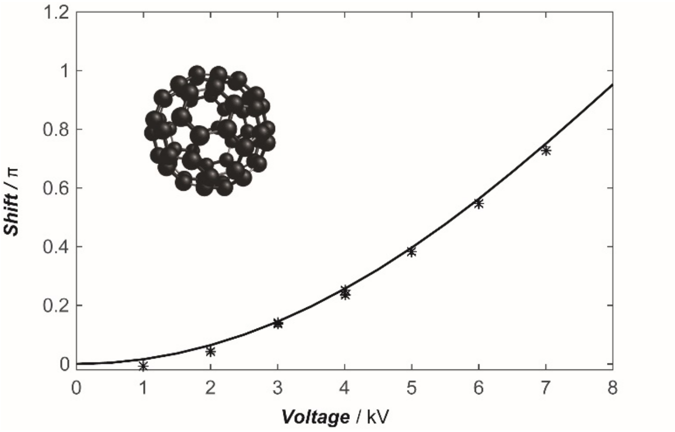

Figure S2. Determination of the geometric scaling factor *K* using a quadratic fit to the C_{60} matter-wave deflection curve. From a series of such measurements we determine the calibration for *K*.

d) Computation of the electronic molecular properties

In our MD simulations, the molecules were set into a computational box of $100 \times 100 \times 100\ \text{Å}^3$ size using periodic boundary conditions. The DFT calculations were then performed using Gaussian09 using the functionals described in the main text and the default settings with respect to convergence criteria and field strength.

To obtain an ensemble average over an equilibrium thermodynamic quantity in the MD simulation one can, in principle, average over many particles in a single time step, over one particle over all time steps or even over many particles and many time steps. For simplicity, we here assume that ergodicity applies and that these different approaches converge within the limit of experimental accuracy. Improved precision could be obtained by additional simulations with parallel tempering (replica exchange) and the quality of the statistics can be controlled with additional Monte Carlo simulation.

One may further ask whether molecules in high vacuum – as in our experiment – can be rightfully described by CHARMM force fields which have been originally optimized for collective ensembles with non-bonding interactions, i.e. in solvents. The agreement between theory and experiment suggests that this is no issue for phylloquinone or α-tocopherol. The observations for β-carotene are expected to be dominated by the dynamics rather than by the force field. Additional ab initio molecular dynamics (AIMD) simulations support the statement that at high experimental temperatures the classical MD simulations employing the CHARMM force field describe the trajectories in conformational space reasonably well. In the AIMD simulations the nuclear motion is integrated

using the velocity-Verlet algorithm, and the electronic potential is provided by DFT at the PBE0/3-21G level of theory. Since AIMD simulations are computationally expensive, we have here only calculated the first 50 ps using a small basis set for a preliminary comparison. In contrast to our classical MD simulations, the stochastic velocity rescaling thermostat[5] has been used with the same relaxation time of 0.1 ps. The small basis set can be justified since the binding and torsion angles are the relevant geometric parameters in our simulations. They are generally less sensitive to changes in the basis set than bond lengths.

Following the theoretical approaches described in the main text we have simulated the electronic properties of all three (pro)vitamins. Complementary to the data of the main text, here we show the time evolution of the static polarizability and dipole moment of β-carotene and phylloquinone.

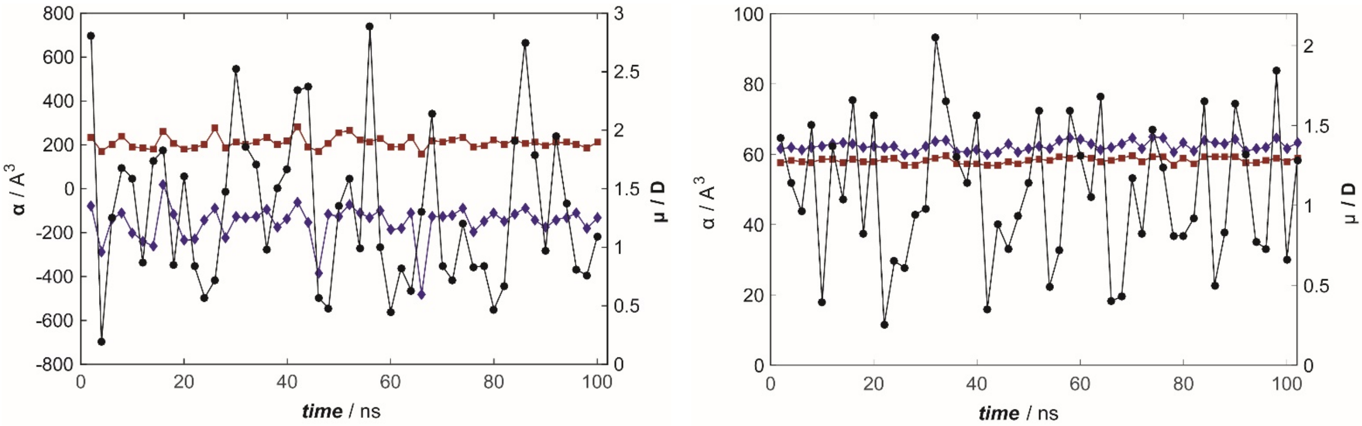

Figure S2. Combined MDS and DFT calculation of the electronic properties of β-carotene (left) and phylloquinone (right). Evolution of their static (α_{stat}, red squares) and optical (α_{opt}(532 nm), blue diamonds) polarizability as well as their electric dipole moment (μ, black circles). Here we only show the simulations at the CAM-B3LYP/Def2TZVP level of theory.

e) Molecular configurations with extreme electronic properties

While the polarizability stays rather constant throughout all thermal evolution, the electric dipole moment can vary by several hundred percent. This is due to the exposure of charge imbalances in the bending process. Figure S3 shows highest and lowest dipole moment configurations together with the dipole moments for β-carotene and phylloquinone. While structural changes are dramatic in case of α-tocopherol and phylloquinone, β-carotene is more rigid. However, due to its inversion symmetry and the corresponding dipole moment of zero in the thermal ground state, the influence of the vibrations is much stronger than in α-tocopherol and phylloquinone.

f) Experimental uncertainties in Table 1

For the polarizability, a systematic contribution results from the necessary scaling of the experimental visibility to the theoretically expected visibility. We consider that the measured susceptibilities depend not only on the deflecting field but also on the assumed interaction of the molecular beam with the light grating, as in our deflection experiments we imprint a shift on an interference pattern that is evolving in free flight. Furthermore, we consider the cross correlation of the uncertainty in the molecules' velocity and the light grating intensity with the calibration factor K.

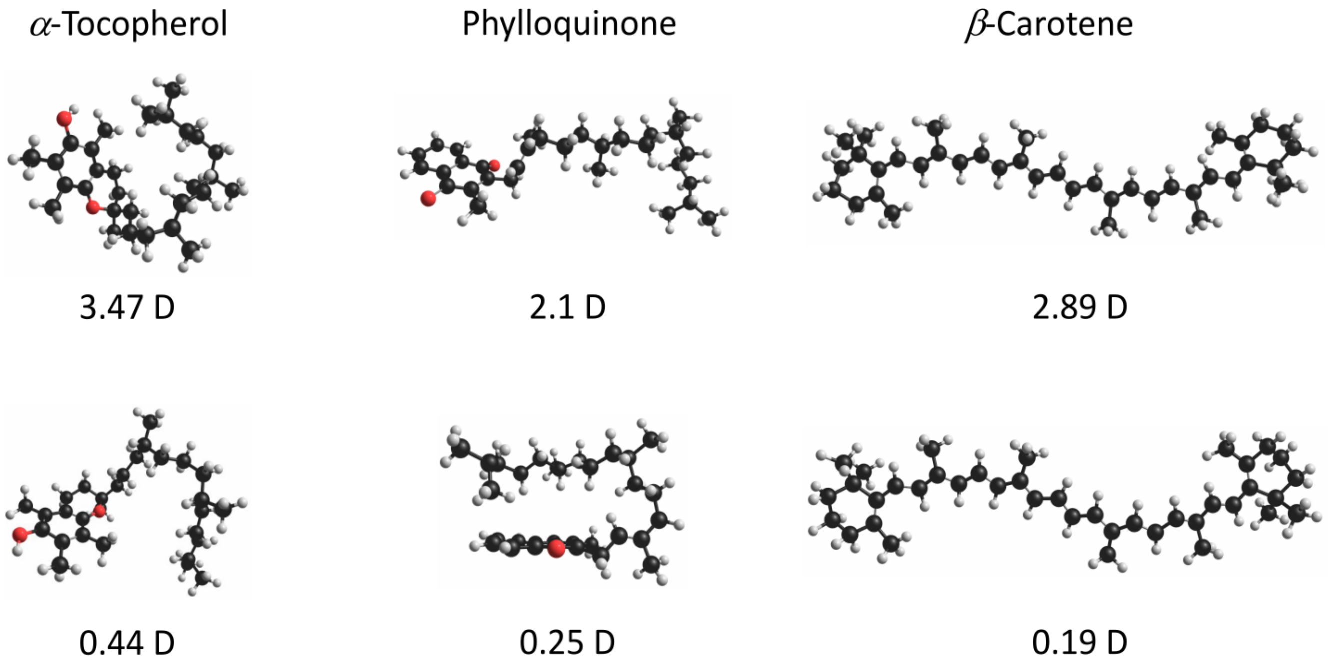

Figure S3: Left: α-tocopherol shows the strongest contribution of fluctuating dipole moments to the total susceptibility in this sequence of vitamins with $\Delta\chi = 4\pi\varepsilon_0 \times 24$ Å^3. **Middle:** In phylloquinone the floppiness is high but the thermal contribution to the thermal average is lowest of all vitamins here with $\Delta\chi = 4\pi\varepsilon_0 \times 7$Å ^{3}. **Right:** β-carotene has the highest polarizability because of its conjugate electron system. The additional thermal contribution to the total susceptibility is $\Delta\chi = 4\pi\varepsilon_0 \times 12$ Å^3.

References

1. A. Stibor, K. Hornberger, L. Hackermüller, A. Zeilinger and M. Arndt, Laser Physics **15** (1), 10-17 (2005).
2. L. Hackermüller, K. Hornberger, B. Brezger, A. Zeilinger and M. Arndt, Nature **427** (6976), 711-714 (2004).
3. K. Hornberger, S. Uttenthaler, B. Brezger, L. Hackermüller, M. Arndt and A. Zeilinger, Phys. Rev. Lett. **90**, 160401 (2003).
4. K. Hornberger, S. Gerlich, H. Ulbricht, L. Hackermüller, S. Nimmrichter, I. Goldt, O. Boltalina and M. Arndt, New J. Phys. **11**, 043032 (2009).
5. G. Bussi, D. Donadio and M. Parrinello, J. Chem. Phys. **126** (1), 014101 (2007).

a) Quelle und Detektor

Die Vitamine wurden in einem Keramikofen mit einer Öffnung von 0.2 x 2 mm verdampft bzw. sublimiert. Mit einer Füllung von etwa 200 mg konnten wir für etwa vier Stunden mit stabilem Signal messen. Die Geschwindigkeit des emittierten Molekülstrahls ist annähernd Maxwell-Boltzmann verteilt, mit leichtem Versatz zu höheren Geschwindigkeiten. Bei Temperaturen von 400 - 450 K lag die wahrscheinlichste Geschwindigkeit bei etwa 200 m/s.

Zur Bestimmung der Geschwindigkeitsverteilung zerteilt ein *Random Choppe*r den Molekülstrahl in eine pseudozufällige Paketsequenz, deren Flugzeit im QMS zeitaufgelöst detektiert wird. Der *Chopper* besteht aus einer Scheibe von 64 Öffnungen, die sich mit 15 Hz dreht. Daraus ergibt sich eine effektive Frequenz von 980 Hz.

Die Moleküle werden durch Elektronenstoß mit einer Energie von 70 eV ionisiert und im Quadrupol-Massenspektrometer gezählt (QMS Extrel CMS). Zur Erhöhung der Zählrate wird das QMS bei reduzierter Massenauflösung und die interne Konversionsdynode bei 9 kV betrieben. Fragmente, die im Ofen oder bei der Ionisation entstehen, werden im QMS aussortiert und nur intakte Moleküle registriert.

b) Optische Polarisierbarkeiten

Kalibration der Strahltaille des Lasers

Zur korrekten Interpretation der V(P) Kurven, muss man die Laserintensität in G_2 genau kennen. In Abbildung 3 reicht die Laserleistung reicht aus, das erste Maximum der V(P) Kurve für β-Carotin zu erreichen. Die gilt auch für α-Tocopherol, nicht aber für Phyllochinon, Aufgrund seiner geringeren Polarisierbarkeit.

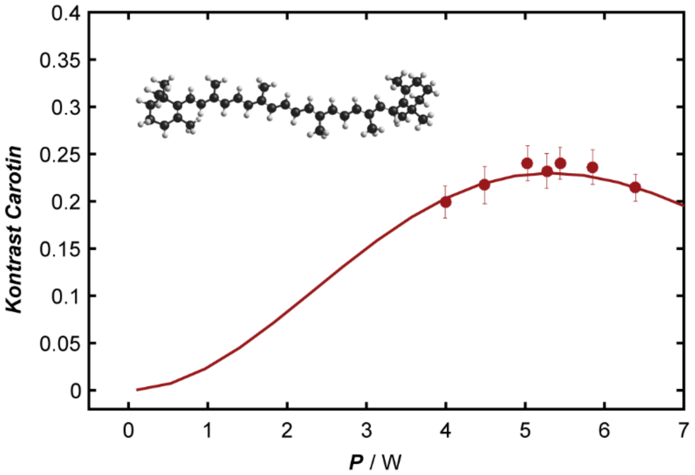

Abbildung S1.

Interferenzkontrast von β −Carotin als Funktion der Leistung des Gitterlasers in G_2. Aufgrund einer geänderten Laserjustage ist der Maximalkontrast hier mit 23% etwas niedriger als in Abbildung 2 des Haupttextes.

Laserleistung und Strahlweite wurden außerhalb der Kammer mit 5% Genauigkeit bestimmt, bevor der Strahl im Vakuum durch eine Zylinderlinse von f=100 mm Fokuslänge auf den Interferometerspiegel fokussiert wurde. Variationen in der optischen Polarisierbarkeit und Laserleistung verschieben die Kurve horizontal. Die Höhe des Interferenzkontrasts kann verringert werden durch Absorption von Photonen aus dem Gitter in G_2, aber auch durch unvollständige Interferometerjustage, technisches Rauschen[3], thermische[4] oder Stoß-Dekohärenz[5]. Um diese Effekte zu quantifizieren, haben wir eine allfällige Kontrastreduktion sowie die Strahlparameter[6] in G_2 mit Hilfe der gut bekannten C_{60} Moleküle kalibriert. Dazu haben wir den experimentellen Interferenzkontrast auf ein experimentell bestätigtes theoretisches Modell normiert[1]. Aus der horizontalen Position des Maximums der *V(P)* Kurve für C_{60} errechnen wir die vertikale Strahltaille des Lichtgitters. Mit einer Polarisierbarkeit von C_{60} von $\alpha_{opt} = 4\pi\varepsilon_0 \times 87(10)\,\text{Å}^3$ und einem optischen Absorptionsquerschnitt $\sigma_{opt}(532nm) = 2.9 \times 10^{-18} cm^2$

erhalten wir für die Messungen mit β-Carotin eine vertikale Strahlweite von 440 µm, während sie für alle anderen Vitamine in späteren Experimenten 920 µm betrug.

c) Elektrische Suszeptibilität

Messung der Ablenkung des Interferenzmusters

Wir finden das Interferenzmuster durch Verfahren des dritten Gitters quer über den Molekülstrahl. Um die feldabhängige Verschiebung des Molekülstrahls zu bestimmen, werden im selben Gitterscan zwei Interferenzbilder aufgenommen, eines für die Referenzspannung von 1 kV, das zweite für die jeweilige Ablenkspannung. An jeder Position des dritten Gitters G_3 nehmen wir die Zählraten für die beiden Spannungen auf. Wir haben in steigender und fallender Spannungsfolge gemessen, um potentielle systematische Fehler zu identifizieren.

Kalibrierung des elektrischen Ablenkfeldes

Der Proportionalitätsfaktor K in unseren Ablenkmessungen hängt von der Elektrodengeometrie und ihrer Lage zum Molekülstrahl ab. Er kann mit einem Molekül bekannter Polarisierbarkeit bestimmt werden. Wir verwenden wieder C_{60} und bestimmen K=1924(78) für identische experimentelle Bedingungen wie bei den Messungen für die Vitamine K und E (siehe Figur S1). Der Plot zeigt die Ablenkung des Interferenzmusters in Einheiten der „Fringe-Phase“ $\Delta\varphi = 2\pi \cdot \Delta x/d$. Ein Versatz von π entspricht der halben Periode des Interferograms.

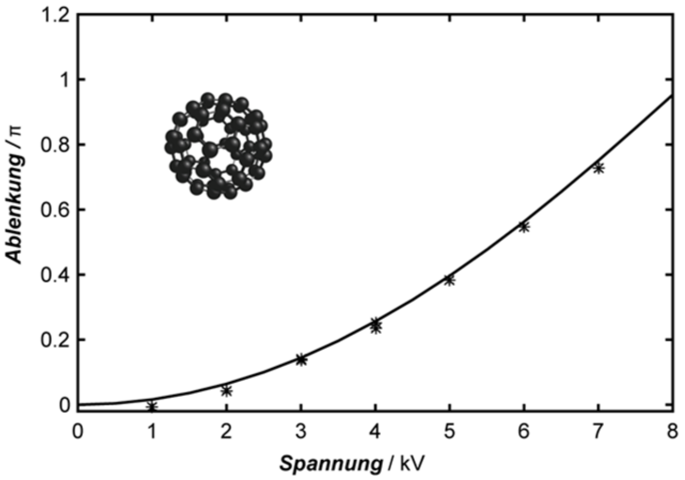

Abbildung S2:

Bestimmung des geometrischen Faktors K aus dem quadratischen Fit an die Ablenkkurve des Fullerens C_{60}.

d) Berechnung der elektronischen Moleküleigenschaften

In unseren MD Simulationen wurden die Moleküle in einer Box von $100 \times 100 \times 100\,\text{Å}^3$ platziert. Die DFT Rechnungen wurden mit Gaussian 09 unter Verwendung der im Haupttext diskutierten Funktionale berechnet. Dabei wurden die Standardeinstellungen für die Konvergenzkriterien und die Feldstärke verwendet. Um in der MD Simulation das Ensemblemittel thermodynamischen Gleichgewicht zu erhalten, kann man wie folgt verfahren: (i) Man kann über alle Teilchen in einem einzigen Zeitschritt mitteln (ii) für ein Teilchen über alle Zeitschritte oder (iii) über viele Teilchen über viele Zeitschritte. Wir nehmen hier an, dass das Ergoden-Theorem erfüllt ist und dass diese drei Ansätze für ein System im Gleichgewicht und ausreichend viele Schritte oder Teilchen konvergieren. Zusätzliche Simulationen mit „replica exchange“ würden die Präzision erhöhen und die Qualität der Statistik kann mit Monte Carlo Simulationen geprüft werden.

Man kann auch fragen, ob CHARMM Kraftfelder zur Beschreibung isolierter Moleküle im Hochvakuum geeignet sind, da sie ursprünglich für kollektive Ensembles mit nicht-bindenden Wechselwirkungen in Lösungen optimiert wurden. Die gute Übereinstimmung zwischen Theorie und Experiment deutet darauf hin, dass die Kraftfelder für Phyllochinon oder α-Tocopherol gut sind und die Rechnungen für β-Carotin scheinen eher von der molekularen

Isomerisierung und Dynamik als von den Kraftfeldern dominiert zu werden. Zusätzliche *ab initio* Moleküldynamik (AIMD) Simulationen zeigen, dass die klassischen MD Simulationen unter Verwendung des CHARMM Kraftfeldes bei den hohen experimentellen Temperaturen die Trajektorien im Konformationsraum hinreichend gut beschreiben. In den AIMD Simulationen wird die Kernbewegung mit dem Geschwindigkeits-Verlet Algorithmus integriert und das elektronische Potential auf dem PBE0/3-21G Niveau der Theorie bestimmt. Für einen ersten Vergleich haben wir hier nur die ersten 50 ps unter Verwendung eines kleinen Basissatzes berechnet, da AIMD Simulationen sehr viel Rechenzeit benötigen. Im Gegensatz zu unseren klassischen MD Simulationen wurde der stochastische Geschwindigkeits-Reskalierungs-Thermostat mit einer Relaxationszeit von 0.1 ps verwendet. Der kleine Basissatz scheint gerechtfertigt, da die relevanten geometrischen Parameter der Simulation, also die Bindungs- und Torsionswinkel im Allgemeinen weniger stark von der Variation des Basissatzes beeinflusst werden als die Bindungslänge.

Mit den im Haupttext beschriebenen Ansätze haben wir die elektronischen Eigenschaften aller drei (Pro)Vitamine simuliert. Der Vollständigkeit halber zeigen wir in Abbildung S3 noch die errechnete zeitliche Entwicklung der statischen Polarisierbarkeit und des Dipolmoments von Phyllochinon und β-Carotin.

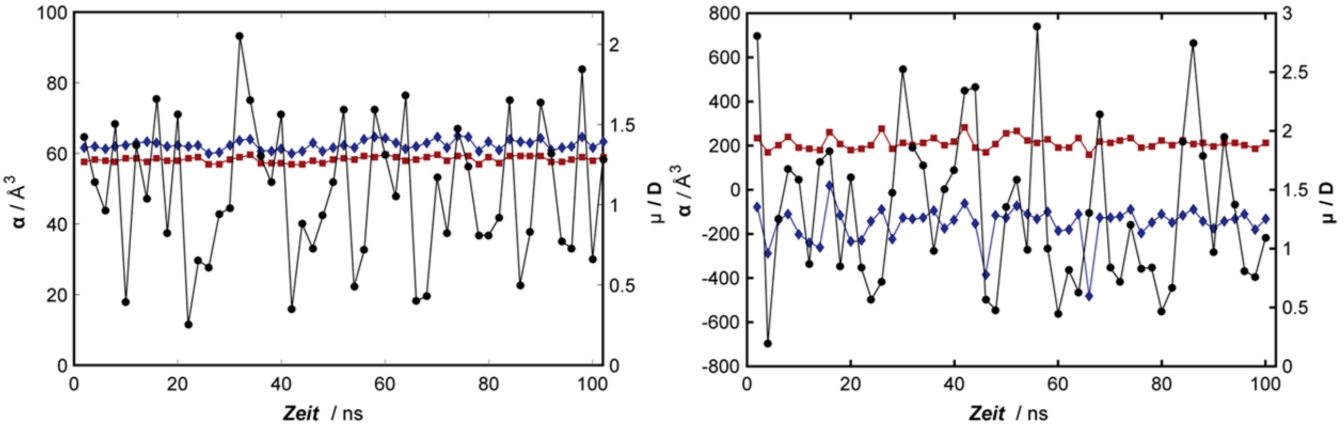

Abbildung S3. *Kombinierte MD und DFT Simulation der elektronischen Eigenschaften von Phyllochinon (links) und β-Carotin (rechts). Wir zeigen die Entwicklung ihrer statischen (rote Quadrate) und optischen (532 nm) Polarisierbarkeit (blaue Diamanten) sowie den Betrag ihres elektrischen Dipolmoments (μ, schwarze Kreise). Diese Simulationen basieren auf dem CAM-B3LYP/Def2TZVP Theorielevel.*

e) Molekulare Konfigurationen mit extremen elektronischen Eigenschaften

Während die thermischen Fluktuationen keinen großen Einfluss auf die Polarisierbarkeit haben, ändert sich das elektrische Dipolmoment um mehrere hundert Prozent. Dies liegt daran, dass bei der Verzerrung der Struktur Ladungsungleichgewichte auftreten. Abbildung S4 zeigt die Konfigurationen mit den höchsten und niedrigsten Dipolmomenten zusammen mit den entsprechenden Dipolmomenten für alle untersuchten Moleküle. Während im Fall von *α*-Tocopherol and Phyllochinon große strukturelle Änderungen auftreten, erweist sich β-Carotin als steifer. Aufgrund seiner Inversionssymmetrie hat β-Carotin im thermischen Grundzustand kein Dipolmoment. Vibrationen haben hier aber einen wesentlich stärkeren Einfluss als bei *α*-Tocopherol und Phyllochinon.

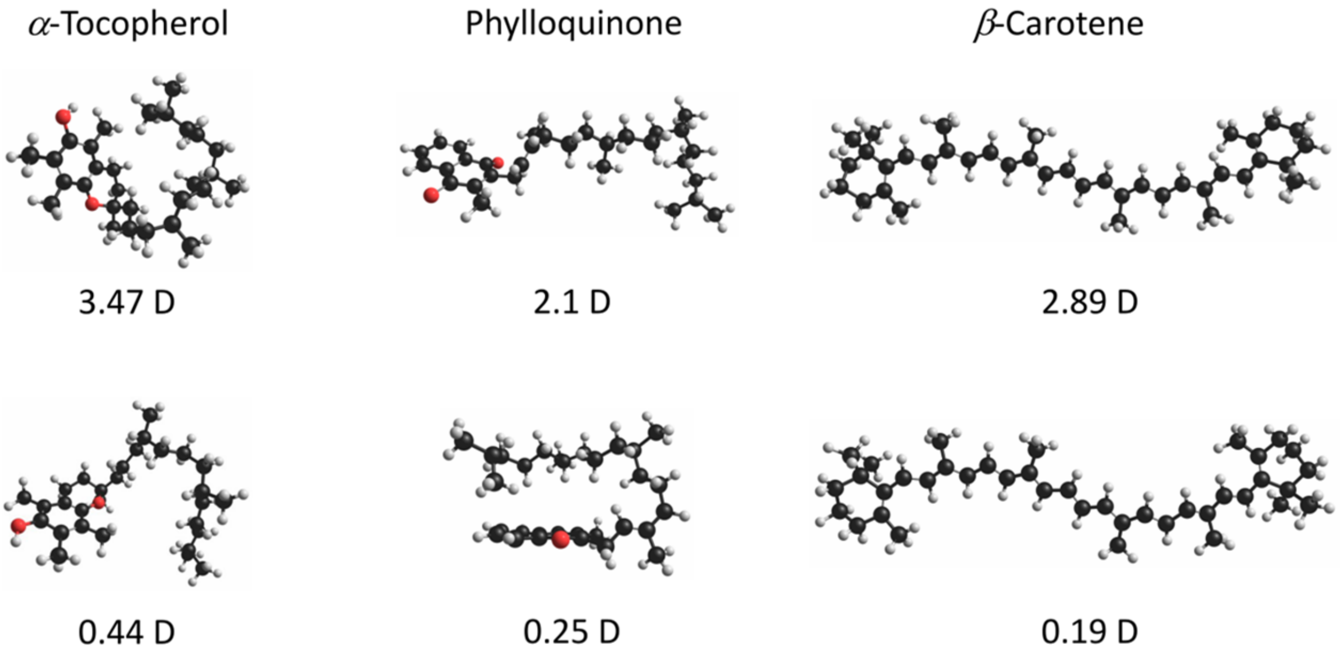

Abbildung S4. Links: *α-Tocopherol weist mit* $\Delta\chi = 4\pi\varepsilon_0 \times 24\ \text{Å}^3$ *den stärksten Beitrag des fluktuierenden Dipolmoments zur Gesamtsuszeptibilität auf.* ***Mitte:*** *Das Kerngerüst von Phyllochinon ist sehr beweglich, aber der gemittelte thermische Beitrag ist mit* $\Delta\chi = 4\pi\varepsilon_0 \times 7\ \text{Å}^3$ *der geringste aller drei untersuchten Vitamine.* ***Rechts:*** *Aufgrund seines vollständig konjugierten elektronischen Systems weist β-Carotin die höchste Suszeptibilität auf. Der thermische Beitrag dazu beträgt* $\Delta\chi = 4\pi\varepsilon_0 \times 12\ \text{Å}^3$.

f) Experimentelle Unsicherheiten in Tabelle 1

Die Skalierung des experimentell bestimmten Interferenzkontrasts auf den theoretischen Wert (nach Kalibrierung mit C_{60}) beinhaltet einen systematischen Fehler bei der Bestimmung der Polarisierbarkeit. Wir berücksichtigen weiters, dass die gemessene Suszeptibilität nicht nur von dem angelegten elektrischen Feld abhängt, sondern auch von der Wechselwirkung des Molekülstrahls mit dem Lichtgitter, da wir in den Ablenkexperimenten die Lageverschiebung der molekularen Interferenzstreifen bestimmen, deren Amplitude durch das Lichtgitter beeinflusst wird. Zusätzlich gibt es so auch eine Korrelation zwischen der Unsicherheit in der Kenntnis der Molekülgeschwindigkeit und der Lichtgitterintensität. Dies wird beim Einfluss des experimentell bestimmten Kalibrationsfaktor K berücksichtigt.

Literaturverweise:

1. A. Stibor, K. Hornberger, L. Hackermüller, A. Zeilinger and M. Arndt, Laser Physics 15(1), 10-17 (2005).
2. L. Hackermüller, K. Hornberger, B. Brezger, A. Zeilinger and M. Arndt, Nature 427(6976), 711-714 (2004).
3. K. Hornberger, S. Uttenthaler, B. Brezger, L. Hackermüller, M. Arndt and A. Zeilinger, Phys. Rev. Lett. 90, 160401 (2003).
4. K. Hornberger, S. Gerlich, H. Ulbricht, L. Hackermüller, S. Nimmrichter, I. Goldt, O. Boltalina and M. Arndt, New J. Phys. 11, 043032 (2009).
5. G. Bussi, D. Donadio and M. Parrinello, J. Chem. Phys. 126(1), 014101 (2007).
